# Unique CD44 intronic SNP is associated with tumor grade in breast cancer: a case control study and in silico analysis

**DOI:** 10.1186/s12935-018-0522-2

**Published:** 2018-02-23

**Authors:** Rezvan Esmaeili, Nasrin Abdoli, Fatemeh Yadegari, Mohamadreza Neishaboury, Leila Farahmand, Ahmad Kaviani, Keivan Majidzadeh-A

**Affiliations:** 1 Genetics Department, Breast Cancer Research Center, Motamed Cancer Institute, ACECR, No 146, South Gandhi Ave, Vanak Sq., Tehran, Iran; 2 Azad University of Karaj, Tehran, Iran; 3 Present Address: Department of Surgery, Tehran University of Medical Sciences, No 136, Molla-Sadra St., Tehran, Iran

**Keywords:** CD44, Mutation, Single nucleotide polymorphism, Splice variants, SR proteins, In silico analysis

## Abstract

**Background:**

CD44 encoded by a single gene is a cell surface transmembrane glycoprotein. Exon 2 is one of the important exons to bind CD44 protein to hyaluronan. Experimental evidences show that hyaluronan–CD44 interaction intensifies the proliferation, migration, and invasion of breast cancer cells. Therefore, the current study aimed at investigating the association between specific polymorphisms in exon 2 and its flanking region of CD44 with predisposition to breast cancer.

**Methods:**

In the current study, 175 Iranian female patients with breast cancer and 175 age-matched healthy controls were recruited in biobank, Breast Cancer Research Center, Tehran, Iran. Single nucleotide polymorphisms of CD44 exon 2 and its flanking were analyzed via polymerase chain reaction and gene sequencing techniques. Association between the observed variation with breast cancer risk and clinico-pathological characteristics were studied. Subsequently, bioinformatics analysis was conducted to predict potential exonic splicing enhancer (ESE) motifs changed as the result of a mutation.

**Results:**

A unique polymorphism of the gene encoding CD44 was identified at position 14 nucleotide upstream of exon 2 (A37692→G) by the sequencing method. The A > G polymorphism exhibited a significant association with higher-grades of breast cancer, although no significant relation was found between this polymorphism and breast cancer risk. Finally, computational analysis revealed that the intronic mutation generated a new consensus-binding motif for the splicing factor, SC35, within intron 1.

**Conclusions:**

The current study results indicated that A > G polymorphism was associated with breast cancer development; in addition, in silico analysis with ESE finder prediction software showed that the change created a new SC35 binding site.

## Background

Breast cancer is one of the leading causes of cancer-related mortality and the most prevalent cancer in women worldwide [1]. Family history of the disease is one of the most important risk factors for breast cancer [2, 3]. Therefore, inherited genetic makeup may bring about the utmost risk of developing cancer. The most important familial breast cancer susceptibility genes are *BRCA1* and *BRCA2*, which are inherited with autosomal dominant pattern. The carriers of mutant genes have a significantly greater risk for developing breast and ovary cancers [4, 5]. According to the current estimates, known breast cancer susceptibility genes are responsible for only less than 25% of the familial clustering of breast cancer [6]. This fact requires the execution of further studies to detect other responsible genes.

One of the interesting candidates of such genes is *CD44*, which plays pivotal roles in normal and pathologic processes of the body. *CD44* is composed of 20 exons spanning about 50 kb of DNA [7]. *CD44* is one of the most notable examples of alternative splicing because 10 of a total of 20 exons can be included or skipped to produce over 1000 potential isoforms [8]. The 1st and the last 5 exons of the gene are constant exons [9]. The protein is involved in normal processes of the body such as cell–cell and cell–extracellular matrix interactions, and thereby, plays important roles in lymphocyte migration, extravasation and homing, T cell and B-cell adhesion, T-cell signaling, and apoptosis [2, 10–16]. Some studies point out its qualitative and quantitative expression changes in breast cancer. There is a noticeable link between *CD44* expression and breast cancer aggressiveness [17–19]. The altered splicing patterns are reported in many cancer-related genes, including *FGFR*, *CD44*, *MDM2*, *IIp45*, etc. [20]. Serine/arginine-rich (SR) proteins implicated as trans-acting factors are closely correlated with tumorigenesis. The SR family of proteins is bound to ESEs; therefore, they can promote exon recognition and correct splicing. Nucleotide substitution in ESEs can affect the binding of SR proteins to these ESEs, leading to splicing errors and exon skipping or intron retention [21].

High level of SR proteins expression is correlated with a progression from a pre-neoplastic to metastatic cancer in a mouse model of mammary cancer [22]. Increased expression of the SR proteins is also associated with an increased complexity of CD44 isoforms, indicating increased SR protein expression that may promote alternative splicing of CD44 mRNA and contribute to tumor progression [23]. CD44 is also one of the well-known markers of breast cancer-initiating cells (BCIC). These cells are phenotypically distinguished cells, which account for the development of primary and metastatic tumor. CD44 acts as a BCIC biomarker and the main contributor to BCICs maintenance, activity, drug, and radiation resistance, as well as pre-metastatic niche preparation [10, 16, 19]. Exon 2 is one of the important exons to bind CD44 protein to hyaluronan [24]. Experimental evidence shows that hyaluronan–CD44 interaction intensifies the proliferation, migration, invasion, tumor angiogenesis, and patient survival of breast cancer cells. This polymorphism was examined in a white and African–American population and there was a significant relationship between the frequency of this polymorphism and the risk of breast cancer. Based on our knowledge, there are no studies reporting the association between CD44 polymorphisms and breast cancer risk as well as clinico-pathological properties in Iranian patients. Therefore, the current study aimed at investigating the association between specific polymorphisms in exon 2 and its flanking region with breast cancer risk.

## Materials and methods

### Study population

In the current case–control study, 350 subjects (175 cases and 175 age-matched controls) were investigated. To prepare the subjects, blood samples were taken from them; then, the specimens were sent to the Breast Cancer Research Institute (BCRC-BB) and collected in the Biological Bank of the institute. The inclusion criteria of the study were: having breast cancer and the availability of blood sample. Healthy matched controls were selected from age-matched females. They did not have any signs or familial history of breast cancer. BCRC-BB is obliged to ethical guidelines and recommendations for biobanks on the storage and use of human biological samples. The current study was approved by the BCRC Research Ethics Committee and performed according to the ethical standards as laid down in the 1964 Declaration of Helsinki. In addition, all patients provided written informed consent before entering the biobank. The diagnosis of cancer was confirmed by histopathological analysis. Clinical information such as stage, grade, hormonal receptor status (ER, PR, and Her2), tumor size, and clinical lymph node was obtained from the hospital records.

### Genomic DNA isolation and PCR amplification

Genomic DNA was extracted from peripheral blood samples using the genomic DNA purification kit (Promega, Madison, USA) according to the manufacturer’s recommendations. DNA sequencing method was used to determine the polymorphisms in the *CD44* exon 2 (166 bp) and its flanking region (271 bp). Briefly, genomic DNA from each sample was amplified using 0.5 µM forward (5′-CCGGCCTTATTTGACTTTTTAAGGAGTCTG-3′) and reveres (5′-CTCCAGTTGTCATACAGGTTGCA GATTGAC-3′) primers designed by Zhou et al. [25]. The PCR program was 94 °C for 5 min (1 cycle), 94 °C for 40 s, 64 °C for 30 s, and 72 °C for 35 s (30 cycles), and the final extension at 72 °C for 5 min. The PCR product, with an expected length of 437 bp, was analyzed in 2% gel agarose electrophoresis. Then, the PCR products were sequenced by standard methods using BigDye terminator DNA sequencing kit (Applied Biosystems, Foster City, CA) with *CD44* forward and reverse primers. Sequences were blasted and then, DNA Baser software was used to investigate the sequencing results [26].

### Statistical analysis

Statistical analysis was performed with SPSS version 18.0 software. Associations between the single-nucleotide polymorphism (SNP) and breast cancer risk were assessed using the Chi square test. A 2-tailed P value < 0.05 was considered statistically significant.

### In silico analysis

In order to identify the potential impact of the A > G variant on the efficiency of splicing, in silico analyses were performed using Human Splicing Finder version 3.0 (HSF) and ESE finder with mutant and reference sequences [27, 28].

HSF was used to predict acceptor (3′ ss) and donor (5′ ss) splice sites strength based on position weight matrices.

ESEs were recognized by individual SR proteins in the study subjects. ESE finder was used to identify ESE motifs that changed as a result of a mutation (ESE-finder: http://rulai.cshl.edu/tools/ESE/). The default threshold values were considered to identify sites responsible for 4 SR proteins, including alternative splicing factor/splicing factor2 (ASF/SF2), SR splicing factor 5 (SRp40), SR splicing factor 3 (SC35), and SR splicing factor 6 (SRp55). Only the wild type or mutant sequence motifs with scores higher than or equal to the threshold were considered.

## Results

### Characteristics of participants

The demographic and clinico-pathological characteristics of the case and control groups were summarized in Table 1. There was no statistical difference between the case and control groups concerning age distribution.

**Table 1:** Demographic features of patients

Characteristics	Cases
Age (years)	
Mean ± SD	48.14 ± 11.586
Histology	
Ductal	83.2
Lobular	9.8
Mixed	4.8
Missing data	2.2
Stage^a^	
1 and 2	29.5
3 and 4	18.8
Missing data	51.7
Grade	
I	9.2
II	38.1
III	23.3
Missing data	32.4
Clinical tumor size (cm)	
0	0.8
< 2	34.4
2.1–5	47.5
> 5	17.2
Missing data	32.4
Clinical lymph node	
0	14.2
< 3	9.7
4 and 9	12.5
> 9	5.1
Missing data	58.5
Her2 expression	
Positive	22.2
2+	5.1
Negative	42.6
Missing data	30.1
ER expression	
Positive	47.2
Negative	22.7
Missing data	30.1
PR expression	
Positive	44.3
Negative	25.6
Missing data	30.1

^a^Stage grouping are based on American Joint Committee on Cancer (AJCC), Estrogen receptor (ER), Progesterone receptor (PR), Her2/neu are based on IHC results. Cut point of positivity is based on American Society of Clinical Oncology (ASCO) guideline for IHC

### Distribution of polymorphisms in exon 2 and its flanking of *CD44*

**Fig. 1:**
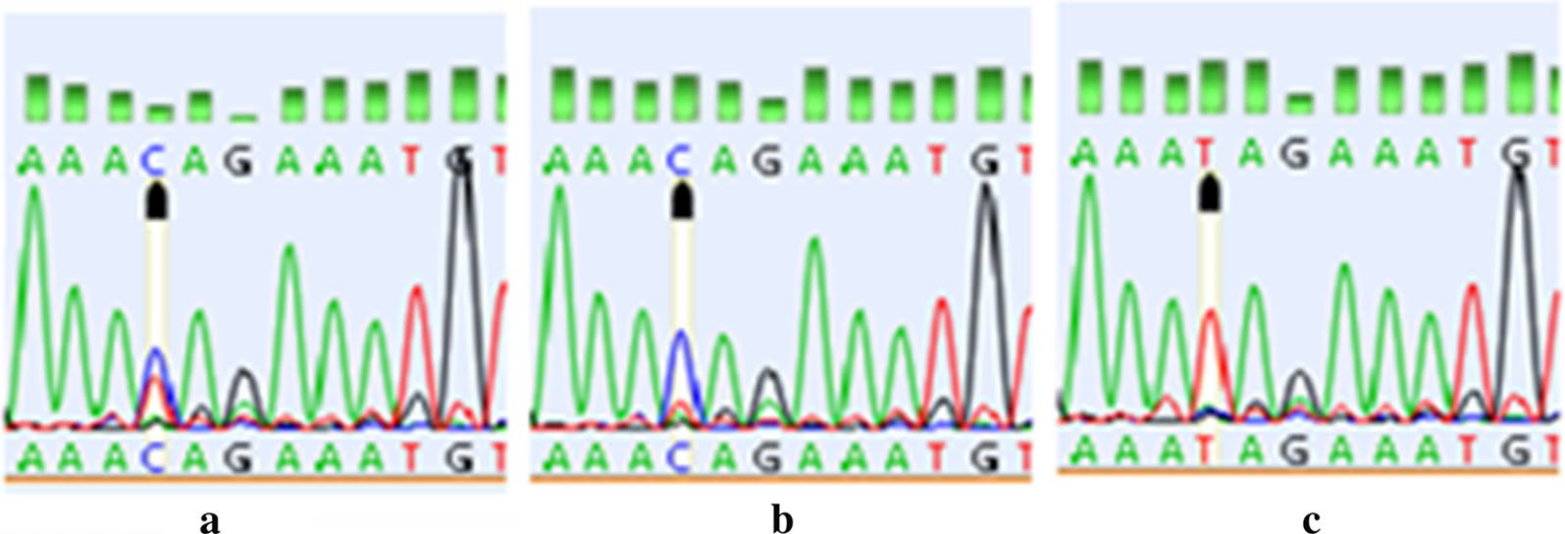
Distributions of polymorphisms in exon 2 and its flanking in *CD44.* Chromatogram of A > G heterozygous variant (**a**), homozygous change (**b**), and wild-type sequence (**c**) in *CD44* intron 1, 14 nucleotides upstream of exon 2

Sequence analysis showed a unique polymorphism between *CD44* exon 2 and its upstream intron (intron 1). The polymorphic change was A > G located 14 nucleotides upstream of the exon 2 (A37692→G) (Fig. 1). Result of the Chi square test showed that frequency of A > G variant was 42.6 and 36.9% in patients and controls, respectively. Accordingly, this polymorphism was higher in females with breast cancer in comparison with that of control population, though this difference was statistically insignificant (P = 0.27). The risk of breast cancer related to *CD44* polymorphism was further examined with stratification by age, family history of breast cancer, pathological type, clinical stage, estrogen/progesterone receptor status (ER, PR), and *Her2* expression. Results of the current study showed no significant associations between the homo- and heterozygous polymorphism and age, ER/PR/HER2 status, or molecular subtype. Interestingly, significant association was found between A > G polymorphism with higher-grade tumors (grade 3) [P = 0.009, odds ratio (OR) = 2.5], indicating that A > G polymorphism increased approximately twice more the risk of higher grade of breast cancer (Table 2). Polymorphism in intron 1 *CD44* was identified in 58.69% of females with breast cancer grade 3. Another A > G nucleotide change was detected in intron 1 that was a 37-nucleotide upstream exon 2 (A37669→G) in only two patients with breast cancer. Both heterozygous and homozygous SNPs were identified in patients with breast cancer.

**Table 2:** Association of *CD44* polymorphism with tumor grade

Genotype	Grade 1 and 2 N (%)	Grade 3	Odd ratio	P value
AG/GG	34 (35.4%)	27 (58.7%)	2.5	0.009
AA	62 (64.6%)	19 (41.3%)		

### In silico analysis

The disruption of cis-elements or the change in splice site strength are among the approaches in which mutations can affect splicing. These effects were investigated by Human Splicing Finder version 3.0 and ESE-finder, respectively [27, 28].

ESE finder showed that mutation generated a new SC35 binding site within intron 1. This sequence is shown in boldface in Table 3 and the mutated nucleotide is underlined. The score for the newly generated SC35 binding motif was higher than the defaults threshold. Human Splicing Finder version 3.0 revealed that polymorphism does not alter an authentic splice site (data not shown).

**Table 3:** The prediction of the connected ESE motifs by examining the intron 1 and exon 2 sequences with the ESE finder program

SR protein type	SR-protein binding motifs found in control sequence	SR-protein binding motifs found in patient sequence
SC35	–	CATTTCTG

## Discussion

The *CD44* gene is located on human chromosome 11p13 in which at least 10 internal exons can be alternatively spliced [7]. The CD44 protein binds to hyaluronan via regions coded by the exons 2 and 5 [24]. Experimental evidence shows that hyaluronan–CD44 interaction intensifies the proliferation, migration, and invasion of breast cancer cells. It is also associated with tumor angiogenesis and patient survival [29–33]. There are few studies reporting the association between *CD44* polymorphisms and breast cancer risk [25, 34–37]. This polymorphism was examined in a white and African–American population and there was a significant relationship between the frequency of this polymorphism and the risk of breast cancer. It is also known that patients with breast cancer also had a significantly higher percentage of unique SNP than normal donors [25]. This polymorphism probably leads to tumor progression. It is reported that *CD44* rs353639 variant may associate with breast cancer prognosis; however, both the rs13347 and rs353639 polymorphisms did not affect breast cancer risk in a North Indian population [36]. Zhou et al., also showed that the combined effects of 4 SNPs in *CD44* exon 2 were significantly associated with breast cancer development; however, no significant difference was found between SNPs and breast cancer risk [37]. These reports motivated the authors to investigate the association of specific polymorphisms in exon 2 and its flanking region with breast cancer risk.

In the current study, a significant association was found between A > G polymorphism and higher grades of breast cancer (grade 3), while no significant difference was observed between the said polymorphism and breast cancer risk. To the authors’ best knowledge, the association between A > G polymorphism and cancer grade was reported for the first time in the current study.

AA genotypes are directly proportional to low grade tumors (G 1/2), while this trend is reversed in patients carrying AG/GG genotypes as they showed a significantly higher incidence of grade 3. These data indicate that AG/GG genotypes may lead to higher grades of breast cancer. In another study, a significant association was observed between the same *CD44* polymorphism and breast cancer risk in the population, European descent and African–American. However, there were statistically significant differences in the distribution of allele frequency and breast cancer risk based on ethnic groups; the difference was especially great in the case of the European descent (P < 0.005). The difference in allele frequencies between above-mentioned study and our data may be due to the variations in sample selection criteria, geographical distribution, and different ethnic groups. It was reported that African–American have higher mortality rate due to breast cancer than the European descent; however, African–American females had a lower incidence of breast cancer compared with European females [38]. Zhou et al., also showed that frequency of the unique SNP in *CD44* intron 1 was higher both in African–American patients with breast cancer and normal donors than white population, while breast cancer was diagnosed at an earlier age in African–American females carrying the unique polymorphism in intron 1 than the European females. These results were consistent with those of the current study that showed the association of the unique SNP in intron 1 with breast cancer invasion and mortality [25].

It is estimated that 15% of point mutations causing human genetic diseases disrupt splice sites or splicing control sequences [39]. Most mutations result in exon skipping due to disruption of 5′ and 3′ splice sites. As previously mentioned, since SR proteins usually induce splicing as a result of their connection to ESE and ISE, the ESE Finder program was used to examine the presence of potential SR protein binding sites within the exon 2 of *CD44* and its flanking region [27]. Remarkably, this analysis identified a new high-affinity SC35 binding site located within mutant, but not in the wild type sequence. Additionally, SR protein levels can affect *CD44* alternative splicing implicated in tumor progression [40]. In human disease genes, mutations in the ESE sequences linked to aberrant transcripts. For example, a disease-related point mutation at position 26 of the intron of the dE1-α PDH gene activates cryptic 5′ splice site due to increased SC35 binding to an ESE; this activation is consistent with the ESE score values predicted by ESE finder [41]. Based on the in silico analysis, A > G polymorphism within exon 2 flanking region of CD44 may be effective on CD44 splicing due to the creation of a new position for SR proteins. The intronic mutation generates a new binding motif for the SC35 and may lead to part of the intron 1 sequence be incorporated into exon. Further studies are needed to confirm our hypothesis by performing in vitro splicing assays.

The current study had some limitations that should be acknowledged. Since the new alternative splicing site was found in this study, further experimental validation of this site and detecting probable protein varient producing from this site is recommended. In addition, independent studies in other ethnic populations are still needed to confirm our present findings.

## Conclusions

The current study found that A > G polymorphism in intron 1 of *CD44* was associated with higher grades of breast cancer, and generated a new SC35 binding site as predicted by the ESE finder. Since *CD44* alternative splicing and overexpression implicated in tumor progression, more experiments should be performed to determine the probable effects of A > G polymorphism on *CD44* expression in patients with breast cancer.

## Abbreviations

PCR: polymerase chain reaction
ESE: exonic splicing enhancer
SR: serine/arginine-rich
BCIC: breast cancer-initiating cells
BCRC-BB: Breast Cancer Research Institute
SNP: single-nucleotide polymorphism
HSF: Human Splicing Finder version
ASF/SF2: alternative splicing factor/splicing factor2
ER/PR: estrogen/progesterone receptor status

## References

[1] Kamangar F, Dores GM, Anderson WF (2006). Patterns of cancer incidence, mortality, and prevalence across five continents: defining priorities to reduce cancer disparities in different geographic regions of the world. J Clin Oncol.

[2] Sellers TA (1997). Genetic factors in the pathogenesis of breast cancer: their role and relative importance. J Nutr.

[3] Oskouee MA, Shahmahmoudi S, Nategh R, Esmaeili H-A, Safaeyan F, Moghaddam MZ (2015). Three common TP53 polymorphisms and the risk of breast cancer among groups of Iranian women. Arch Breast Cancer.

[4] Antoniou A, Easton D (2006). Models of genetic susceptibility to breast cancer. Oncogene.

[5] King M-C, Marks JH, Mandell JB (2003). Breast and ovarian cancer risks due to inherited mutations in BRCA1 and BRCA2. Science.

[6] Thompson D, Easton D (2004). The genetic epidemiology of breast cancer genes. J Mammary Gland Biol Neoplasia.

[7] Goodfellow PN, Banting G, Wiles MV, Tunnacliffe A, Parkar M, Solomon E (1982). The gene, MIC4, which controls expression of the antigen defined by monoclonal antibody F10. 44.2, is on human chromosome 11. Eur J Immunol.

[8] Bell MV, Cowper AE, Lefranc M-P, Bell JI, Screaton GR (1998). Influence of intron length on alternative splicing of CD44. Mol Cell Biol.

[9] Screaton GR, Bell MV, Jackson DG, Cornelis FB, Gerth U, Bell JI (1992). Genomic structure of DNA encoding the lymphocyte homing receptor CD44 reveals at least 12 alternatively spliced exons. Proc Natl Acad Sci.

[10] Al-Hajj M, Wicha MS, Benito-Hernandez A, Morrison SJ, Clarke MF (2003). Prospective identification of tumorigenic breast cancer cells. Proc Natl Acad Sci.

[11] Bourguignon L, Zhu D, Zhu H (1998). CD44 isoform–cytoskeleton interaction in oncogenic signaling and tumor progression. Front Biosci.

[12] Chen D, McKallip RJ, Zeytun A, Do Y, Lombard C, Robertson JL (2001). CD44-deficient mice exhibit enhanced hepatitis after concanavalin A injection: evidence for involvement of CD44 in activation-induced cell death. J Immunol.

[13] McKallip RJ, Fisher M, Do Y, Szakal AK, Gunthert U, Nagarkatti PS (2003). Targeted deletion of CD44v7 exon leads to decreased endothelial cell injury but not tumor cell killing mediated by interleukin-2-activated cytolytic lymphocytes. J Biol Chem.

[14] McKallip RJ, Fisher M, Gunthert U, Szakal AK, Nagarkatti PS, Nagarkatti M (2005). Role of CD44 and its v7 isoform in staphylococcal enterotoxin B-induced toxic shock: CD44 deficiency on hepatic mononuclear cells leads to reduced activation-induced apoptosis that results in increased liver damage. Infect Immun.

[15] Rafi A, Nagarkatti M, Nagarkatti PS (1997). Hyaluronate–CD44 interactions can induce murine B-cell activation. Blood.

[16] Sales KM, Winslet MC, Seifalian AM (2007). Stem cells and cancer: an overview. Stem Cell Rev.

[17] Bankfalvi A, Terpe HJ, Breukelmann D, Bier B, Rempe D, Pschadka G (1998). Gains and losses of CD44 expression during breast carcinogenesis and tumour progression. Histopathology.

[18] Kaufmann M, von Minckwitz G, Heider K, Ponta H, Herrlich P, Sinn H (1995). CD44 variant exon epitopes in primary breast cancer and length of survival. Lancet.

[19] Dall P, Heider KH, Sinn HP, Skroch-Angel P, Adolf G, Kaufmann M (1995). Comparison of immunohistochemistry and RT-PCR for detection of CD44v-expression, a new prognostic factor in human breast cancer. Int J Cancer.

[20] Srebrow A, Kornblihtt AR (2006). The connection between splicing and cancer. J Cell Sci.

[21] Cartegni L, Chew SL, Krainer AR (2002). Listening to silence and understanding nonsense: exonic mutations that affect splicing. Nat Rev Genet.

[22] Stickeler E, Kittrell F, Medina D, Berget SM (1999). Stage-specific changes in SR splicing factors and alternative splicing in mammary tumorigenesis. Oncogene.

[23] Günthert U, Hofmann M, Rudy W, Reber S, Zöller M, Haußmann I (1991). A new variant of glycoprotein CD44 confers metastatic potential to rat carcinoma cells. Cell.

[24] Telen MJ, Udani M, Washington MK, Levesque MC, Lloyd E, Rao N (1996). A blood group-related polymorphism of CD44 abolishes a hyaluronan-binding consensus sequence without preventing hyaluronan binding. J Biol Chem.

[25] Zhou J, Nagarkatti PS, Zhong Y, Creek K, Zhang J, Nagarkatti M (2010). Unique SNP in CD44 intron 1 and its role in breast cancer development. Anticancer Res.

[26] DNA Sequence Assembler v4. Heracle BioSoft; 2013. http://www.DnaBaser.com.

[27] Cartegni L, Wang J, Zhu Z, Zhang MQ, Krainer AR (2003). ESEfinder: a web resource to identify exonic splicing enhancers. Nucleic Acids Res.

[28] Desmet F-O, Hamroun D, Lalande M, Collod-Béroud G, Claustres M, Béroud C (2009). Human Splicing Finder: an online bioinformatics tool to predict splicing signals. Nucleic Acids Res.

[29] Catterall JBGM, Turner GA (1995). Hyaluronic acid, cell adhesion and metastasis. Cancer J.

[30] Knudson CB, Knudson W (1993). Hyaluronan-binding proteins in development, tissue homeostasis, and disease. FASEB J.

[31] Kosaki R, Watanabe K, Yamaguchi Y (1999). Overproduction of hyaluronan by expression of the hyaluronan synthase Has2 enhances anchorage-independent growth and tumorigenicity. Cancer Res.

[32] Naor D, Sionov RV, Ish-Shalom D (1997). CD44: structure, function and association with the malignant process. Adv Cancer Res.

[33] So JY, Lee HJ, Smolarek AK, Paul S, Wang C-X, Maehr H (2011). A novel Gemini vitamin D analog represses the expression of a stem cell marker CD44 in breast cancer. Mol Pharmacol.

[34] Jiang L, Deng J, Zhu X, Zheng J, You Y, Li N (2012). CD44 rs13347 C > T polymorphism predicts breast cancer risk and prognosis in Chinese populations. Breast Cancer Res.

[35] Zhou X, Wu C (2012). Association of CD44 polymorphisms with chemosensitivity to anthracycline-based chemotherapy in breast cancer. J Jilin Univ.

[36] Tulsyan S, Agarwal G, Lal P, Agrawal S, Mittal RD, Mittal B (2013). CD44 gene polymorphisms in breast cancer risk and prognosis: a study in North Indian population. PLoS ONE.

[37] Zhou J, Nagarkatti PS, Zhong Y, Zhang J, Nagarkatti M (2011). Implications of single nucleotide polymorphisms in CD44 exon 2 in risk for breast cancer. Eur J Cancer Prev Off J Eur Cancer Prev Org (ECP).

[38] Jemal A, Siegel R, Ward E, Hao Y, Xu J, Murray T (2008). Cancer statistics, 2008. CA Cancer J Clin.

[39] Krawczak M, Reiss J, Cooper DN (1992). The mutational spectrum of single base-pair substitutions in mRNA splice junctions of human genes: causes and consequences. Hum Genet.

[40] Götte M, Yip GW (2006). Heparanase, hyaluronan, and CD44 in cancers: a breast carcinoma perspective. Cancer Res.

[41] Miné M, Brivet M, Touati G, Grabowski P, Abitbol M, Marsac C (2003). Splicing error in E1α pyruvate dehydrogenase mRNA caused by novel intronic mutation responsible for lactic acidosis and mental retardation. J Biol Chem.

